# Killing by Contraction

**Published:** 1875-12

**Authors:** 


### KILLING BY CONTRACTION.

It is singular that, in the manifold styles of dress which succeed each other with the rapidity of almost barometrical variations of temperature, not one of them favors the freedom of the thorax or chest. That is the axle to which all pieces are attached, and the pivot round which they revolve, if at all. A small waist is the first consideration. It is, therefore, the study of those who conceive they are too large just where there should be no interference with the respiratory apparatus, how to diminish their diameter. This desideratum has been the premature death of thousands upon thousands of the fairest and most promising young ladies before they had time to learn the dangers they were inviting, by following the example of those who teach by their practice that they prefer conformity to the requirements of a perverted taste, to exemption from the penalties of being out of shape, in the sense of those who exercise no judgment in regard to this important matter.

The smaller the waist, therefore, the better, provided there is space enough preserved for descent of food to the stomach. Stays are instrumentalities for staying the development of the chest. Beginning early, the ribs are pinioned closely, and by unrelaxing ligation—the lacing being carried to the last endurable point without arresting respiration—their growth is arrested to an extent only familiarly known to anatomists. Their function is nearly destroyed, as they become anchylosed, or welded, where they were intended to have motion up and down, according to the inflation and collapse of the lungs. After being subjected to the torture of stays—for such it is, however eloquently those who have lived through the operation of having their chests kept down to the capacity of a child of twelve years may argue to the contrary—breathing with them is an abridged function, or it may be arrested without hesitation. They have counteracted nature, and in various ways must, and do, suffer in consequence. Thoracic compression alters the form of the lungs.

The chest is naturally broad at the base, becoming narrower at the top—a cone-shaped structure. Women make it narrow where it should be broad, and broader at the apex, where it was originally narrow. The lowest air-cells of the lobes cannot expand when air is inhaled, while those in the upper region of the lungs are preternaturally put upon the stretch, in order to provide surface for the creation of blood. One end of each lung is compelled to do more toward maintaining life than it was organized to do, while the lower part is prevented from giving much more than a feeble degree of assistance. Favored, as many robust women are, with a fine organization in other respects, they can live out a long life in comparative health and comfort; but they are few, when compared to the vast number who fall short and die before they have attained all they might have had on earth.

